# Research progress of tsRNAs in kidney diseases

**DOI:** 10.7717/peerj.20315

**Published:** 2025-11-10

**Authors:** Jialing Wang, Yanzhe Wang, Fengqin Li, Xinmiao Xie, Xinyue Chen, Tong Wu, Xiaoxia Wang

**Affiliations:** 1 Department of Nephrology, Tongren Hospital, School of Medicine, Shanghai Jiao Tong University, Shanghai, China; 2 Bengbu Medical University, Anhui, China

**Keywords:** TsRNAs, TRFs, TiRNAs, Kidney diseases, Biological function

## Abstract

Transfer RNA-derived small RNAs (tsRNAs) are a class of regulatory non-coding RNAs generated through enzymatic cleavage of precursor or mature tRNAs. In recent years, tsRNAs have garnered growing interest in nephrology due to their diverse biological functions and potential clinical significance. This review summarizes current research on the roles of tsRNAs in kidney diseases, including their involvement in gene expression regulation, signal transduction, apoptosis, and inflammation-related pathways. We further highlight their emerging mechanistic contributions in conditions such as acute kidney injury, chronic kidney disease, and glomerulonephritis. Finally, we discuss the prospects of tsRNAs as novel biomarkers for early diagnosis, prognosis assessment, and therapeutic targeting in renal disorders, aiming to offer new insights into kidney disease pathogenesis and management.

## Introduction

Approximately 10% of adults worldwide currently suffer from chronic kidney disease (CKD), leading to around 1.2 million deaths annually (Kalantar-Zadeh et al., 2021). In Asia, the number of CKD patients is as high as 443 million, with nearly 160 million in China alone (Liyanage et al., 2022). This disease is characterized by its irreversible progression to end-stage renal disease (ESRD), which inevitably results in patient mortality (Kalantar-Zadeh et al., 2021). It is estimated that by 2030, the number of people requiring renal replacement therapy (RRT) will reach 543.9 million worldwide (Liyanage et al., 2015), and by 2040, CKD will become the fifth leading cause of death globally (Foreman et al., 2018). Addressing kidney health in Asian countries, the Global Kidney Health Atlas (GKHA) has highlighted that China, as one of the countries with the highest number of CKD patients, faces a substantial burden (Bharati & Jha, 2022). Recent studies have suggested that transfer RNA-derived small RNAs (tsRNAs) may play a role in kidney diseases by regulating gene expression, influencing protein synthesis, and inhibiting apoptosis (Li et al., 2024). Exploring the pathogenesis of kidney diseases, identifying drug intervention targets, and discovering new biomarkers are critical for advancing research in this field.

This review is aimed at researchers, clinicians, and translational medicine researchers in the fields of nephrology and molecular biology, with the goal of providing a comprehensive reference for exploring the mechanisms of tsRNAs in kidney diseases, developing biomarkers, and promoting translational applications in clinical nephrology.

## Survey Methodology

Literature retrieval was conducted through the PubMed and Web of Science databases using the keyword combination “tsRNAs OR tRFs OR tiRNAs AND kidney disease” combined with Boolean operators to screen for articles up to August 1, 2025. The inclusion criteria were: original experimental, observational, or clinical studies exploring the role of tsRNAs/tRFs/tiRNAs in kidney diseases (such as acute kidney injury, chronic kidney disease, and diabetic nephropathy). Exclusion criteria included: articles outside the time range and duplicate articles, studies not related to tsRNAs/tRFs/tiRNAs, and non-experimental studies (such as meta-analyses, and editorials), as well as studies not related to kidney diseases. Systematic screening was carried out to ensure the focus, originality, and clinical relevance of the research content.

## Definition of tsRNAs

**Figure 1:**
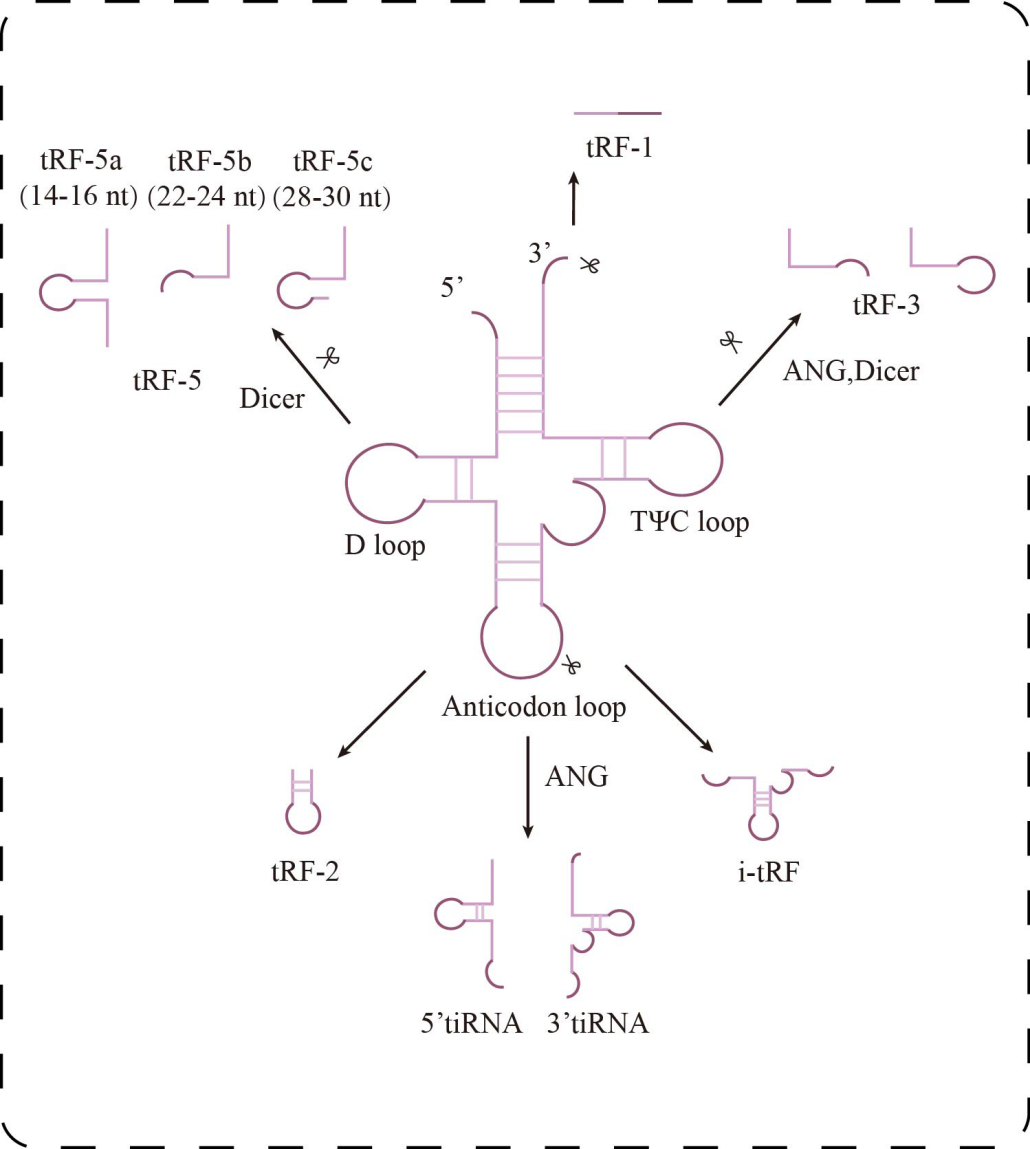
Research progress of tsRNAs in kidney diseases. Classification of tsRNAs. tsRNAs are classified into two major categories: tiRNAs and tRFs. tRFs include: tRF-1 (3′ end sequence of precursor tRNA), tRF-3 (cut at TΨ C loop), tRF-5 (cut at D loop, with a/b/c subtypes), tRF-2 (cut at anticodon loop), and i-tRF (internal fragment). tiRNAs are generated by ANG cleavage and are divided into 5′-tiRNA and 3′-tiRNA.

Transfer RNA-derived small RNAs (tsRNAs) are a group of small non-coding RNAs generated from precursor or mature tRNA, initially discovered in prokaryotes, but increasingly recognized and studied in eukaryotes in recent years (Zuo et al., 2021). Depending on the cleavage site, tsRNAs can be categorized into two main types: tRNA-derived stress-induced RNAs (tiRNAs), which are produced through cleavage by angiogenin and ribonuclease under stress conditions, and tRNA-derived fragments (tRFs), which are generated by nuclear cleavage during either the precursor or mature stage of tRNA. Based on their origin and characteristics, tRFs can be further divided into five subtypes (Fig. 1): tRF-1, tRF-2, tRF-3, tRF-5, and i-tRF (Chu et al., 2022; Lu et al., 2021). tRF-1 is derived from the 3′ trailer sequence of precursor tRNA and contains a poly-U sequence, tRF-3 is generated by cleavage of the TΨC loop of mature tRNA, and tRF-5 is produced by Dicer cleavage of the D-loop of tRNA. tRF-2, on the other hand, consists of a small fragment containing the anticodon of tRNA (Wang et al., 2022). tRF-5 can also be classified into three subtypes based on its length: tRF-5a (14–16 nt), tRF-5b (22–24 nt), and tRF-5c (28–30 nt) (Di Fazio & Gullerova, 2023). tiRNAs, also known as tRNA halves, are divided into two categories: 3′ tiRNA and 5′ tiRNA (Chu et al., 2022).

Recent advances in the detection of tsRNAs have largely relied on two major technological innovations. On the computational side, the tRF2Cancer platform integrates three core modules: tRFfinder, which specializes in identifying small RNA fragments of 18–30 nucleotides, optimal for conventional sequencing; tRFinCancer, which analyzes differential expression in cancer; and tRFBrowser, which maps RNA modification sites (Zheng et al., 2016). This integrative approach has proven effective in exosome research. For example, Zhou et al. (2022) utilized a three-step strategy—aligning to a tRNA reference database, identifying differentially expressed tRFs, and predicting potential functions—to efficiently uncover disease-associated tRFs. On the experimental front, PANDORA-seq introduces a novel enzymatic treatment using T4 PNK and AlkB to remove RNA modifications that typically hinder small RNA sequencing (Shi et al., 2021). While originally developed for oncology studies, this standardized bioinformatics and biochemical workflow offers valuable methodological insight for kidney-related tsRNAs research. At present, the nephrology field lacks a dedicated tsRNAs database comparable to tRF2Cancer. Future research would benefit from developing a platform tailored to kidney disease, integrating expression profiles and functional annotations of tsRNAs. Such a resource could significantly accelerate the transition from correlative observations to mechanistic understanding in tsRNA-based diagnostics and pathophysiological studies.

## Biological Functions of tsRNAs

Rather than representing a single-mode regulatory class, tsRNAs participate in diverse layers of gene regulation, engaging multiple stages of the central dogma to form a highly intricate, multidimensional regulatory network. Accumulating evidence suggests their functional repertoire encompasses at least four core domains: AGO-dependent gene silencing, translational regulation, retrotransposon suppression, and immunometabolic reprogramming. Elucidating these mechanisms and their interconnectivity is critical to understanding the role of tsRNAs in kidney disease pathogenesis.

**Figure 2:**
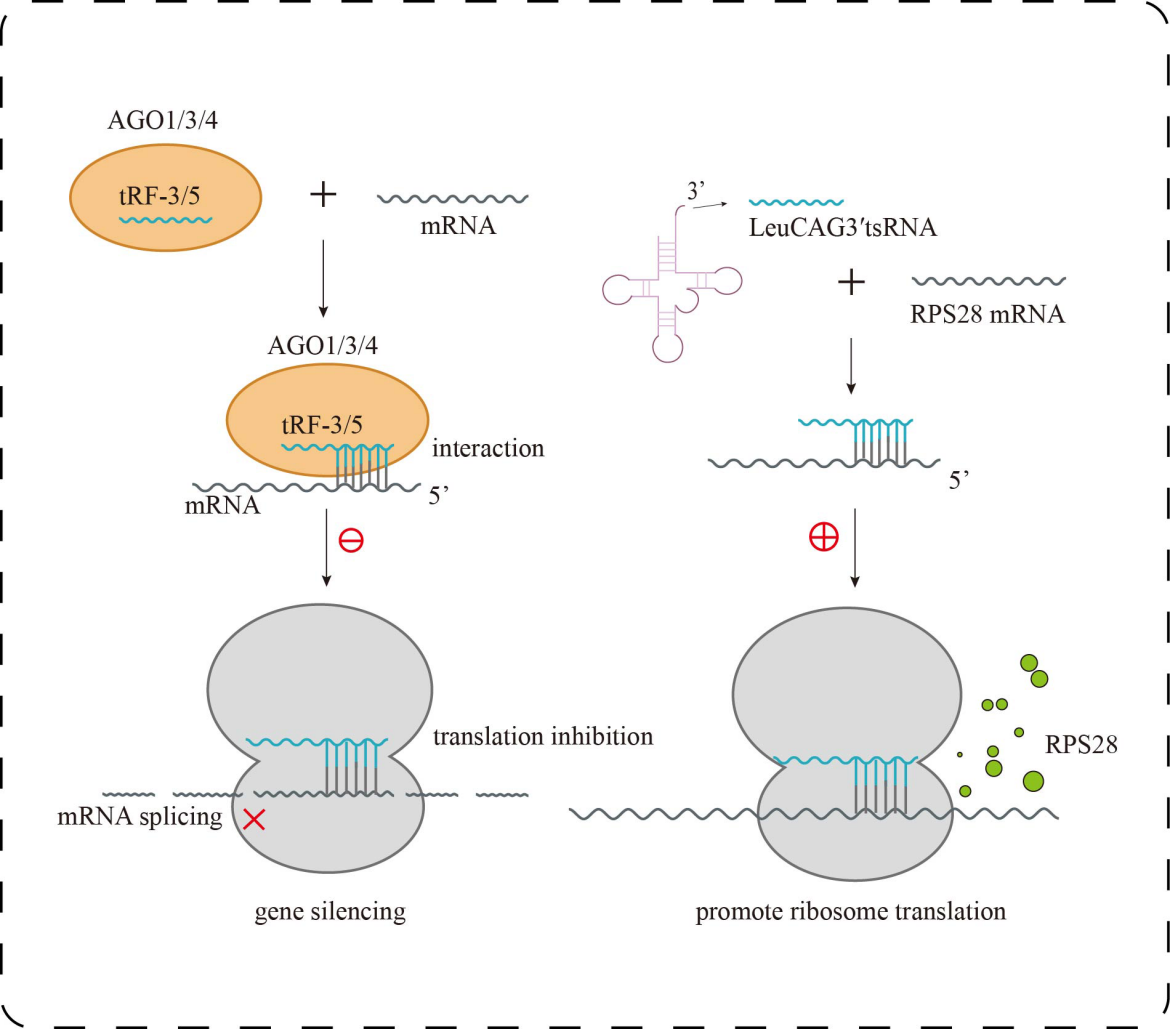
Research progress of tsRNAs in kidney diseases. Biological functions of tsRNAs. tRF-3 or tRF-5 forms a complex with AGO1, AGO3, or AGO4, targeting mRNA to inhibit translation and trigger gene silencing; LeuCAG3 ′ tsRNA binds complementarily to RPS28 mRNA, enhancing translation efficiency and promoting the formation of RPS28 protein.

In human embryonic kidney cells (HEK293), it was found that tRF-3 and tRF-5 exhibit stronger binding affinity to AGO1, AGO3, and AGO4 than to AGO2, the primary effector of canonical miRNA pathways (Fig. 2). Interestingly, tRF-3 and miRNAs share a similar T-to-C mutation pattern at AGO-binding sites, whereas tRF-5 displays a distinct binding profile (Kumar et al., 2014). Moreover, tRF-3b silences target genes *via* AGO-dependent seed pairing, yet this activity is disrupted when m^1^A modification alters its seed sequence, impairing gene repression without affecting AGO association (Su et al., 2022). These findings suggest that tRFs actively engage in RNA interference pathways but do so *via* unique sequence features and modification-dependent mechanisms, expanding the functional complexity beyond canonical miRNA paradigms.

tsRNA-mediated regulation extends beyond the cytoplasm. In HEK293T cells, knockdown of the endoribonuclease Dicer led to a concurrent decrease in both tsRNAs and miRNAs. Additionally, fluorescently labeled tsRNAs predominantly localized to the nucleus in breast cancer cells, whereas traditional siRNAs remained cytoplasmic. Further analysis showed that certain nuclear tsRNAs preferentially bind early intronic regions of nascent transcripts, mediating AGO2-dependent pre-mRNA intron cleavage and silencing (Di Fazio et al., 2022). This Dicer-dependent nuclear RNA interference mechanism, distinct from classical cytoplasmic pathways, suggests a novel strategy for transcriptional regulation and may offer a platform for developing next-generation gene silencing therapeutics. Although tRF^GluTTC^ does not directly mediate DNA methylation or histone modification, it exerts epigenetic-like regulatory effects through post-transcriptional mechanisms. Specifically, it forms a RISC complex *via* AGO binding, targeting the mRNA stability of transcription factors KLF9, KLF11, and KLF12, thereby indirectly modulating adipogenesis-related genes such as PPARγ and C/EBPα (Shen et al., 2019). This indicates that tsRNAs can function at both transcriptional and post-transcriptional levels to orchestrate rapid, systemic gene regulation. In the context of acute kidney injury (AKI), such rapid response systems are likely particularly critical for maintaining cellular integrity under stress.

Under stress conditions, tsRNAs serve as key regulators of protein synthesis homeostasis. The 5′-tiRNA Ala, derived from mature tRNA cleavage, forms a tetramolecular G-quadruplex (RG4) through its 5′ terminal oligo-Guanine motif (5′TOG). This unique structure displaces the eIF4F complex from the 5′ cap of mRNAs in a conformation-dependent manner, effectively inhibiting cap-dependent translation initiation and leading to global translational arrest. Additionally, pseudouridine-modified tsRNAs (mTOG-Ψ) can disrupt the formation of the PABPC1–PAIP1 complex and downregulate key components of the ribosome such as RPL23, RPL29, and the assembly factor EIF6, directly interfering with ribosome biogenesis (Guzzi et al., 2022). In an *in vivo* hepatocellular carcinoma model, LeuCAG3′tsRNA was shown to enhance the translation of ribosomal protein S28 (RPS28) mRNA, promoting cell proliferation, whereas its inhibition reduced RPS28 expression, impaired ribosome assembly, and induced apoptosis (Kim et al., 2017) (Fig. 2). These findings demonstrate that tsRNAs can exert bidirectional control over protein synthesis—globally repressing translation under stress while selectively enhancing the production of survival-related proteins. In chronic kidney diseases such as diabetic nephropathy, such fine-tuned translational control may influence podocyte integrity and ECM deposition.

In embryonic and trophoblast stem cells lacking H3K9 trimethylation, 3′CCA tRFs play a pivotal role in the suppression of endogenous retroviruses (ERVs). The 18-nt isoform directly inhibits reverse transcription by spatially blocking primer binding sites (PBS) on retrotransposons, while the 22-nt isoform loads into RISC to mediate post-transcriptional degradation of ERV transcripts (Schorn et al., 2017). This dual mechanism—targeting the PBS region of LTR elements at both transcriptional and post-transcriptional levels—highlights the evolutionary conservation of tsRNA-mediated transposon defense, which is essential for maintaining genomic stability.

Together, tsRNAs impact both the transcriptome and proteome through a multifunctional network characterized by overlapping and synergistic modules. However, several critical challenges remain. First, the cell-type specificity of tsRNAs subtypes in the kidney—such as in tubular epithelial cells, podocytes, and mesangial cells—requires further delineation. Second, the functional consequences of diverse RNA modifications on tsRNAs activity remain poorly understood. Lastly, future studies should investigate how these pathways intersect under pathophysiological conditions, such as hypoxia, hyperglycemia, or inflammation. In such contexts, tsRNA-regulated signaling may engage in complex cross-talk and dynamic feedback loops, shaping the progression of kidney disease.

## Role of tsRNAs in Acute Kidney Injury

Acute kidney injury (AKI) is a condition characterized by rapid loss of kidney function, manifested by sudden decreases in urine output or anuria, often accompanied by electrolyte imbalance. This injury is usually caused by ischemia, hypoxia, or nephrotoxic substances (Gaut & Liapis, 2021). In classical AKI models induced by renal ischemia-reperfusion or cisplatin nephrotoxicity (Mami & Pallet, 2018), oxidative stress prompts a conformational shift in tRNA molecules—from a stable folded state to an unfolded form. This structural transition represents an early molecular response to cellular stress, occurring even before the onset of DNA damage or apoptosis. The unfolded tRNAs are then selectively cleaved by endoribonucleases such as angiogenin, producing tsRNAs that are released into peripheral circulation during the subclinical phase of tubular injury. This mechanism was further validated in patients undergoing aortic surgery, where plasma levels of tsRNAs rose sharply shortly after reperfusion. Notably, the kinetics of this increase preceded changes in serum creatinine and elevations in urinary KIM-1, suggesting that tsRNAs may serve as highly sensitive biomarkers of early nephron injury. Unfolded tRNAs and their fragments were also observed in regions proximal to damaged mitochondria, indicating that tsRNAs likely originate from structurally compromised nephron units under stress conditions. The ability of these fragments to maintain stability in the circulation—likely *via* encapsulation in exosomes or binding to RNA-binding proteins—provides a structural basis for their utility as reliable diagnostic markers (Mishima et al., 2014). This body of evidence not only highlights the sensitivity of tsRNAs for early AKI detection but also underscores the regulatory role of tRNA structural dynamics in stress responses. These findings open new avenues for mechanistic studies and the development of targeted interventions in AKI.

**Figure 3:**
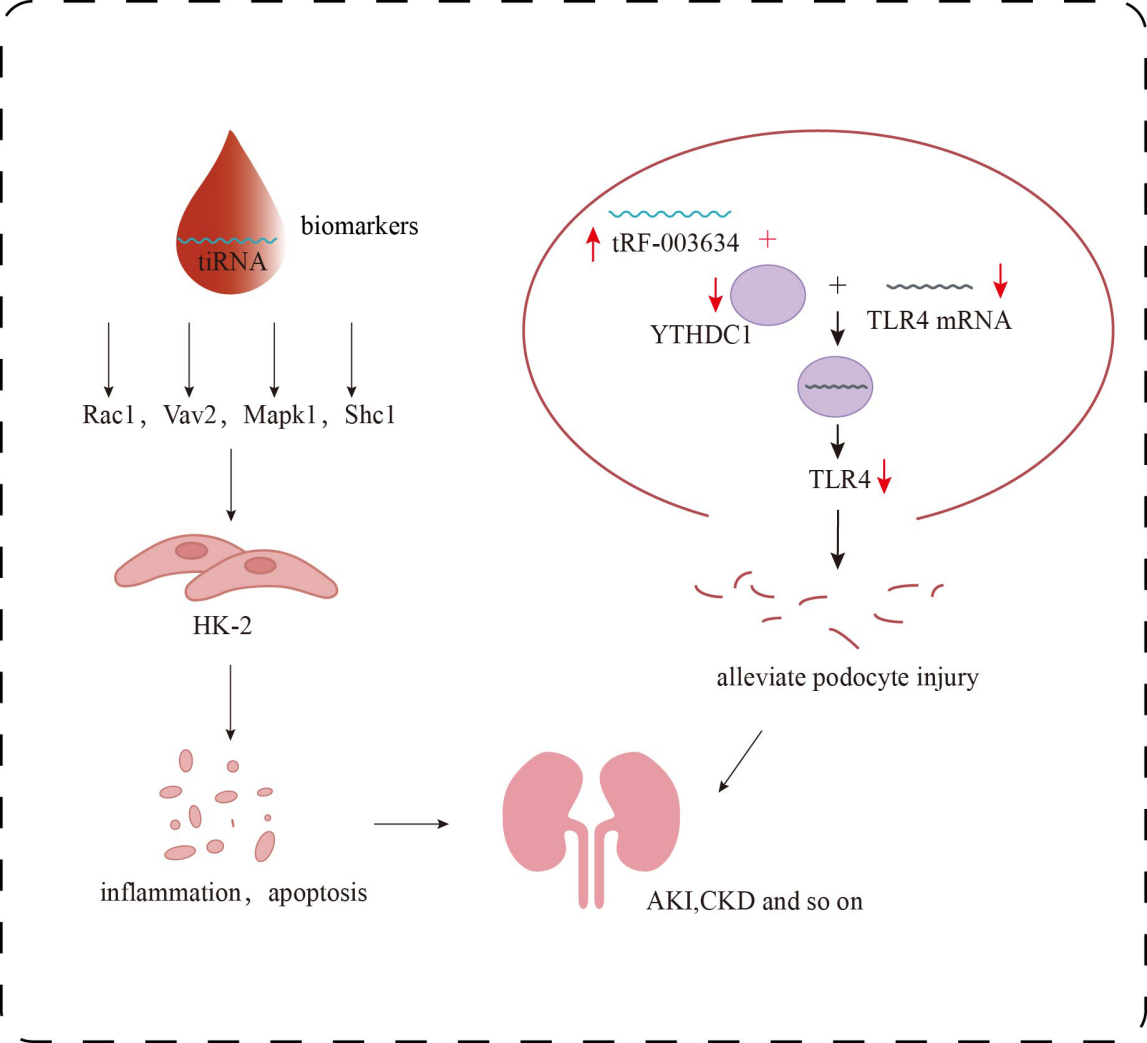
Research progress of tsRNAs in kidney diseases. Role of tsRNAs in kidney disease. The target genes of certain specific tiRNAs, including Rac1, Vav2, Mapk1, and Shc1, are involved in multiple signaling pathways, such as inflammatory responses and apoptosis, and can serve as biomarkers for the detection of early renal damage. tRF-003634 competitively binds to YTHDC1, reducing the stability of TLR4 mRNA and thereby downregulating the expression of TLR4.

Recent research further suggests that tsRNAs function as key regulators within the epitranscriptomic landscape of AKI pathogenesis (Li et al., 2022). By modulating diverse pathways such as immune-inflammatory signaling, metabolic reprogramming, and programmed cell death, tsRNAs critically influence tubular epithelial cell survival and the trajectory of renal recovery. Molecules like tiRNA-Gly-GCC-003 have been shown to exacerbate oxidative stress and apoptosis by affecting natural killer cell cytotoxicity and mitochondrial energy metabolism. Additionally, they regulate the expression of pivotal genes including Rac1 and Mapk1 (Fig. 3), implicating tsRNAs in fibrosis and reperfusion injury–associated signaling networks. These discoveries provide both mechanistic insight and theoretical rationale for tsRNAs as early biomarkers and potential therapeutic targets in AKI.

A groundbreaking study by Li et al. (2025), published in Science, identified a marked upregulation of tRNA-Asp-GTC-3′tDR in murine AKI models. Inhibition of this molecule using antisense oligonucleotides (ASOs) suppressed autophagic activity and exacerbatedtubular necrosis, inflammation, and fibrosis. Conversely, targeted delivery of exogenous tDR *via* cationic polymeric nanoparticles activated autophagy and significantly mitigated renal injury and fibrotic remodeling. Notably, this tDR was also upregulated in kidney tissue from chronic kidney disease (CKD) patients and in the urine of preeclampsia patients with renal complications, showing a strong inverse correlation with histone mRNA expression. These findings underscore the molecule’s central role in RNA-directed autophagy in human kidney diseases and demonstrate its therapeutic potential. The translational value of this tsRNA highlights a pivotal shift in non-coding RNA research—from mechanistic discovery toward clinical application.

## tRFs in chronic kidney disease

### Role of tRFs in diabetic kidney disease

Diabetic Kidney Disease (DKD) is regarded as one of the main causes of CKD in the world, and about 40% of diabetic patients will develop DKD (Alicic, Rooney & Tuttle, 2017). As of 2021, an estimated 5.366 billion adults worldwide were living with diabetes, and this is expected to increase to 7.832 billion by 2045 (Sun et al., 2022). Therefore, early diagnosis and timely intervention can help to delay the development of DKD. As a key factor in the occurrence and development of DKD, podocyte injury can lead to glomerular filtration barrier dysfunction, increase the risk of proteinuria, and then promote the development of DKD. The level of tRFs is separated by the differentiation status of HUPEC cells. For example, tRF-3 expression is up-regulated in APOL1-HR differentiated podocytes, while tRF-1 expression is down-regulated, suggesting that cell proliferation and translational activity are decreased. At the same time, the low expression of leucine and methionine tRFs in undifferentiated and APOL1-HR podocytes indicated that translational activity may be altered depending on differentiation status and APOL1 genotype. These findings were consistent with the results of RNA-seq analysis, revealing a mechanism of translational regulation in podocytes under the influence of differentiation and APOL1 genotype. It is a biological marker of podocyte worthy of further study (Yoshida et al., 2023). However, the HUPEC used may not fully mimic the mechanism of podocyte injury in humans in the *in vitro* differentiation system; therefore, if the *in vitro* differentiation system can be optimized to more closely mimic the mechanism of human podocyte injury, it will be expected to improve the reliability of the study.

In the adriamycin-induced podocyte injury model, Gao et al. (2023) found that the stability of TLR4 mRNA, which may be a downstream target gene of tRF-003634, was decreased by knocking down YTH domain protein 1 (YTHDC1) in podocytes. Overexpression of tRF-003634 also reduced the stability of TLR4 mRNA. These results suggest that tRF-003634 may reduce the stability of TLR4 mRNA by competitively binding to YTHDC1, thereby down-regulating the expression of TLR4 and alleviating podocyte injury *in vitro* and *in vivo*, providing a new strategy for the prevention and treatment of CKD (Fig. 3). In the process of inducing podocyte differentiation *in vitro*, Shi et al. (2020) screened 69 tRFs that were up-regulated and 70 tRFs that were down-regulated. The potential target genes of these differentially expressed tRFs were involved in the regulation of transcription, neovascularization, cell adhesion and other biological processes. These differentially expressed tRFs are related to PI3K-Akt, Rap1, Ras, MAPK, Wnt signaling pathways, which may affect podocyte differentiation. In addition, overexpression of tDR-012842 inhibited the expression of podocyte differentiation markers nephrin and podocin. Fibroblast growth factor 10 (FGF10), one of the putative targets of tDR-012842, was down-regulated in mature podocytes, suggesting that tDR-012842 may promote podocyte differentiation by upregulating FGF10 expression. This finding provides key evidence for the role of tRFs in the regulation of podocyte differentiation. It also points out a new direction for the treatment of chronic kidney disease. However, the detailed mechanism of its action needs to be further explored (Shi et al., 2020). Therefore, in-depth analysis of the role and mechanism of tsRNAs in podocytes will help to discover new targets for DKD treatment and provide more possibilities for delaying the progression of DKD.

In addition, a key feature of diabetic kidney disease is microvascular disease, characterized by glomerulosclerosis and renal tubulointerstitial fibrosis (TIF). Prolonged hyperglycemia stimulates inflammatory responses in the renal tubulointerstitium, leading to excessive accumulation of extracellular matrix (ECM), which in turn promotes TIF development and progression, as well as causing changes in renal hemodynamics, leading to local renal hypoxia and activating fibrotic signaling pathways. Researchers performed high-throughput sequencing on mouse renal tubular epithelial cells treated with high glucose, identifying 27 upregulated and 37 downregulated tRFs. These differentially expressed tRFs were involved in signaling pathways such as autophagy, mTOR, and MAPK. Among them, tRF-1:30-Gln-CTG-4 was significantly downregulated, and its increased transcriptional level helped alleviate high glucose-induced ECM accumulation, suggesting that tRFs may have potential antifibrotic effects. This provides a new perspective on the pathogenesis of diabetic nephropathy and could serve as a new therapeutic target for DN (Ji et al., 2023). However, this study primarily used a mouse *in vitro* model and lacked validation with human kidney tissue samples, necessitating further studies using human samples to confirm the general applicability of the conclusions. Another study found that the expression of tRF3-IleAAT was downregulated in the kidney tissues of db/db mice and in high glucose-induced mouse mesangial cells. By intravenously injecting an adeno-associated virus (AAV) overexpressing tRF3-IleAAT, it was observed that tRF3-IleAAT directly targeted the 3′UTR of its downstream target gene zinc finger protein 281 (ZNF281), thereby inhibiting ferroptosis and ECM synthesis in a DKD mouse model, ultimately improving renal function (Qiao et al., 2024).

### Role of tRFs in mesangial proliferative glomerulonephritis

When the kidney is damaged, mesangial cells release a series of inflammatory mediators, such as TGF-β, which trigger immune responses. Prolonged inflammation and ECM deposition can lead to glomerular fibrosis and sclerosis, resulting in a gradual decline in kidney function (Boi, Ebefors & Nyström, 2023). Therefore, inhibiting mesangial cell proliferation and fibrosis is an important treatment strategy for glomerulonephritis. Studies have reported that treating mesangial cells with transforming growth factor-β1 (TGF-β1) establishes a proliferation model, and high-throughput sequencing was used to compare tRF expression between normal and TGF-β1-treated mesangial cells. The results showed that tDR-000064 and tDR-000103 were significantly downregulated in TGF-β1-treated mesangial cells. GO analysis also revealed that the target gene products of tDR-000064 and tDR-000103 were primarily localized in cytoplasmic microtubules, immune synapses, and cell receptor complexes, and were involved in the positive regulation of the G2/M phase of the cell cycle, suggesting their potential involvement in mesangial cell proliferation. These results indicate that tDR-000064 and tDR-000103 may serve as potential biomarkers and intervention targets for mesangial cell proliferation. However, this study is restricted to transcriptomic profiling and computational prediction. Combining techniques such as CoIP, RNA pull-down, and single-cell sequencing could more comprehensively validate the regulatory effects of tRFs on target genes and their specific mechanisms (Lu et al., 2019).

## Role of tRFs and tiRNAs in Lupus Nephritis

Lupus nephritis (LN) is an autoimmune disease caused by systemic lupus erythematosus (SLE), primarily leading to kidney damage. The severity of the damage depends on the progression of the disease. Early diagnosis and treatment are crucial for this disease. However, current LN diagnosis mainly relies on laboratory tests and kidney biopsy, and there are no specific sensitive biomarkers (Anders et al., 2020; Yu et al., 2022). Studies (Zheng et al., 2022) have found that tsRNA-21109 can inhibit M1 macrophage polarization, while tRF-His-GTG-1 is significantly upregulated in the serum exosomes of SLE patients. Additionally, tRF-3009 is overexpressed in CD4+ T cells, and it is speculated that it may be involved in the pathogenesis of SLE by regulating IFN-α-induced oxidative phosphorylation in CD4+ T cells. Moreover, overexpression of tsRNA-14783 promotes M2 macrophage polarization, which helps alleviate inflammation and promote tissue repair. In LN, the presence of M2c macrophages is often associated with disease progression (Allam et al., 2020; Wang & Hu, 2022). As a subtype of M2 macrophages, whether tsRNA-14783 could influence macrophage function and serve as a marker for LN diagnosis, or how it could regulate LN progression, remains to be studied. Therefore, tsRNAs have demonstrated potential value in SLE diagnosis and treatment by regulating immune cell function and participating in disease pathogenesis.

Another study found that the expression level of tRF-Ala-AGC-2-M4 was significantly elevated in the serum of LN patients compared to healthy individuals, and this elevation correlated with disease activity. Moreover, tRF-Ala-AGC-2-M4 did not show significant differences in other autoimmune diseases, suggesting high specificity. Bioinformatic analysis indicated that tRF-Ala-AGC-2-M4 might be involved in glomerulonephritis signaling pathways associated with LN progression (Zhang et al., 2022). Furthermore, tRF-His-GTG-1 was significantly upregulated in the serum of SLE patients but markedly decreased in LN patients. Although the underlying mechanism remains unclear, Yang et al. (2021) speculated that renal damage might cause increased leakage of tRF-His-GTG-1 into the urine. Another study conducted tsRNAs sequencing of urine exosomes from SLE patients with or without LN, revealing that tRF3-Ile-AAT-1 and tiRNA5-Lys-CTT-1 were elevated in the urine exosomes of LN patients. Specifically, tRF3-Ile-AAT-1 might modulate autoimmunity by affecting signaling pathways related to the Th17/Treg balance, while tiRNA5-Lys-CTT-1 might bind to platelet-derived growth factor (PDGF) and contribute to renal fibrosis. These findings suggest that tsRNAs may contribute to LN progression *via* these pathways, indicating that tsRNAs could serve as non-invasive markers for distinguishing SLE patients with or without LN (Chen et al., 2023; Yang et al., 2021). ROC curve analysis demonstrated that tRF-His-GTG-1 exhibited higher sensitivity and specificity compared to current urinary protein markers used to differentiate SLE patients with glomerulonephritis. This study was the first to highlight the clinical potential of serum tsRNAs in identifying SLE patients with glomerulonephritis, providing a valuable reference for future research on serum tsRNAs as novel non-invasive biomarkers (Yang et al., 2021).

Studies conducted in China identified several abnormally expressed tRFs in CD4+ T cells of SLE patients, with tRF-3009 expression showing a positive correlation with the SLE disease activity index, renal damage, and serum IFN-α levels. Transfection of tRF-3009 mimics into CD4+ T cells, followed by IFN-α stimulation, demonstrated that overexpression of tRF-3009 activated SLE-related signaling pathways and induced oxidative phosphorylation in CD4+ T cells, whereas knockdown of tRF-3009 inhibited IFN-α-induced oxidative phosphorylation. These findings suggest that tRF-3009 may contribute to SLE pathogenesis by regulating CD4+ T cell metabolic pathways, thus providing a novel target for SLE treatment (Geng et al., 2021).

## The Role of tsRNAs in Other Kidney Diseases

### Renal Cell Carcinoma (RCC)

Based on the study by Ding et al., 2024) plasma tsRNAs demonstrate dual clinical utility in renal cell carcinoma (RCC). Through small RNA sequencing and multi-cohort validation, several differentially expressed tsRNAs were identified. Among these, tRF-19-DRMD5112 emerged as a top diagnostic biomarker, with an AUC of 0.865 for early-stage RCC (sensitivity: 80.51%, specificity: 81.31%). LASSO regression further confirmed its value as an independent diagnostic indicator (test set AUC: 0.888). Functionally, tRF-28-87R8WP91IE0K was significantly downregulated in RCC tissues, and its overexpression suppressed tumor migration, invasion, and proliferation *in vitro*. However, this tRF was also markedly downregulated in bladder cancer (AUC: 0.79), highlighting the potential challenge of cross-cancer interference. This study not only proposed plasma tsRNAs as highly sensitive liquid biopsy tools, addressing the current gap in RCC diagnostics, but also established a complete translational trajectory from early diagnosis (tRF-19-DRMD5112) to therapeutic targeting (tRF-28-87R8WP91IE0K). Compared to non-specific renal markers such as creatinine and albuminuria, or miRNAs with potential off-target effects, tsRNAs offer distinct molecular advantages in RCC. For instance, they directly target oncogenic pathways involving YBX1, underscoring their functional specificity (Ding et al., 2021). 5′tRNA-Val-ACC is significantly downregulated in clear cell RCC (Zhao et al., 2018), with its tissue-specific expression tightly linked to tumor progression. Its stability in serum further enhances its value as a liquid biopsy marker. Although still in early stages, the mechanistic specificity and detection stability of tsRNAs make them promising candidates for precise, non-invasive RCC diagnostics.

### Kidney transplantation

Due to their rapid response to oxidative stress, tsRNAs are emerging as promising early biomarkers of graft injury in kidney transplantation. Studies have shown that transplantation-related ischemia-reperfusion injury can rapidly induce tRNA conformational changes—events that precede DNA damage and apoptosis (Kuscu et al., 2021). Notably, 30 differentially expressed tsRNAs have been identified in patients with transplant glomerulopathy. Moreover, the terminal metabolic product of tsRNAs, free m1A, has been linked to increased all-cause mortality in kidney transplant recipients. The stable presence of these molecules in exosomes circulating in peripheral blood supports their potential for real-time graft monitoring and prognostic assessment.

### Focal segmental glomerulosclerosis (FSGS)

The APOL1 high-risk genotype, a major genetic contributor to focal segmental glomerulosclerosis (FSGS), has been shown to alter tRF expression profiles in podocytes. A total of 46 differentially expressed tRFs were identified, including significantly downregulated leucine- and methionine-derived tRFs (Yoshida et al., 2023). These may synergize with APOL1 risk alleles by modulating translational activity and ribosome biogenesis, contributing to podocyte dysfunction and FSGS progression. Importantly, similar changes were observed in kidney tissues of FSGS patients even in the absence of overt histological lesions. Compared to healthy controls, 32 differentially expressed 3′-tRFs and 24 5′-tRFs were detected in glomerular and tubulointerstitial compartments—most of which were significantly upregulated (Williams et al., 2022). These findings suggest that tsRNAs dysregulation may represent an early molecular event in FSGS pathogenesis, preceding morphologic damage and potentially participating in stress signaling and translational imbalance. Thus, tsRNAs not only form a central regulatory axis in APOL1-related podocytopathies but may also provide insight into the molecular origins of FSGS. Future research should prioritize the functional validation of key tRFs and characterize their dynamic changes across disease progression to support translational applications.

### IgA nephropathy

In peripheral blood mononuclear cells of IgA nephropathy patients, tsRNAs exhibit extensive differential expression, with 345 dysregulated molecules identified by high-throughput sequencing. qRT-PCR confirmed the upregulation of key tsRNAs such as tRF-Val-AAC-007 and the downregulation of tiRNA-Val-TAC-004, consistent with sequencing results (Luo et al., 2020). Bioinformatic analysis revealed that upregulated tsRNAs primarily target genes involved in nucleic acid metabolism and ion binding, whereas downregulated ones are associated with cellular component regulation and metal ion interactions. These results suggest tsRNAs have both biomarker potential and therapeutic relevance in IgA nephropathy by modulating pathways related to disease progression.

### Diagnostic value of tsRNAs compared to traditional biomarkers

Beyond disease-specific mechanisms, recent evidence also highlights the diagnostic advantages of tsRNAs compared to conventional renal biomarkers. Serum creatinine and eGFR remain the most widely used clinical indicators for evaluating renal function. However, their limitations—such as insensitivity to early kidney injury and susceptibility to extrarenal factors—have prompted increasing interest in discovering novel biomarkers. tsRNAs, owing to their structural stability, disease specificity, and potential pathophysiological relevance, have emerged as promising alternatives, with certain urinary exosomal tsRNAs such as tRF3-Ile-AAT-1, tiRNA5-Lys-CTT-1, and tRF-His-GTG-1 showing superior diagnostic performance even in early-stage or proteinuria-low nephropathies (Chen et al., 2023; Yang et al., 2021).

### Summary of tsRNAs in various kidney diseases

The emerging roles and potential clinical applications of tsRNAs across a range of kidney diseases are summarized in Table 1, which highlights their diverse regulatory mechanisms and their potential utility as diagnostic biomarkers and therapeutic targets.

**Table 1:** Research progress of tsRNAs in kidney diseases. The key roles of tsRNAs in various kidney diseases and their clinical application potential. tsRNAs are involved in the development of diseases such as acute kidney injury, diabeticnephropathy and lupus nephritis through mechanisms such as regulating gene expression, inhibiting protein synthesis and modulating the function of immune cells. Some tsRNAs canserve as non-invasive biomarkers or potential therapeutic targets, providing new directionsfor early diagnosis and intervention strategies.

**Research progress of tsrnas in kidney diseases**				
**Author, Year**	**Specific tsRNAs name**	**Potential kidney diseases involved**	**Functions of tsRNAs**	**Expected clinical significance**
Mami & Pallet (2018)	tiRNA	Acute or chronic kidney injury	Inhibits protein synthesis, enhances gene and transcript expression, offers cytoprotective effects, while excessive stress may lead to programmed cell death	Serves as a non-invasive biomarker for early detection of kidney injury
Li et al. (2022)	tiRNA-Gly-GCC-003	Acute kidney injury	Regulates immune cell activity and mitochondrial energy metabolism, influences oxidative stress levels and cell death-related signaling pathways	Early diagnostic biomarker for AKI and potential therapeutic target
Li et al. (2025)	tRNA-Asp-GTC-3′tDR	Acute or chronic kidney injury	Positively regulates the autophagy process; expression levels directly affect tubular cell survival and the progression of fibrosis	Provides new therapeutic targets for AKI and subsequent fibrosis
Yoshida et al. (2023)	tRF-1, tRF-3, Leu tRF, Met tRF	Diabetic nephropathy	Translation regulation	Biomarkers for podocyte differentiation
Gao et al. (2023)	tRF-003634	Chronic kidney disease	Reduces TLR4 mRNA stability, downregulates TLR4 expression, alleviates podocyte injury	Offers new strategies for CKD prevention and treatment
Shi et al. (2020)	tDR-012842	Chronic kidney disease	Upregulates FGF10 expression, promotes podocyte differentiation	Provides novel strategies for CKD prevention and treatment
Ji et al. (2023)	tRF-1:30-Gln-CTG-4	Diabetic nephropathy	Reduces high glucose-induced extracellular matrix accumulation, potentially anti-fibrotic	Advances understanding of diabetic nephropathy pathogenesis and provides therapeutic targets
Qiao et al. (2024)	tRF3-IleAAT	Diabetic nephropathy	Inhibits ferroptosis and extracellular matrix synthesis	Slows the progression of diabetic nephropathy
Boi, Ebefors & Nyström (2023)	tDR-000064, tDR-000103	Mesangial proliferative glomerulonephritis	Positively regulates the G2/M phase of the cell cycle, possibly linked to mesangial cell proliferation	Potential biomarkers and therapeutic targets for mesangial cell proliferation
Zheng et al. (2022)	tsRNA-21109	Lupus nephritis	Inhibits macrophage M1 polarization	Provides new therapeutic targets for SLE
Zheng et al. (2022)	tRF-3009	Lupus nephritis	Regulates IFN-α-induced oxidative phosphorylation in CD4+ T cells	Offers novel therapeutic targets for SLE
Wang & Hu (2022)	tsRNA-14783	Lupus nephritis	Promotes macrophage M2 polarization	Serves as a biomarker and therapeutic target for LN
Zhang et al. (2022)	tRF-Ala-AGC-2-M4	Lupus nephritis	Involved in cellular response to endoplasmic reticulum stress, influences cell adhesion and signal transduction	Potential diagnostic biomarker and indicator of disease activity in LN
Yang et al. (2021)	tRF-His-GTG-1	Lupus nephritis	Related to immune regulation	Differentiates SLE with or without LN
Chen et al. (2023)	tRF3-Ile-AAT-1	Lupus nephritis	Involved in cell proliferation, differentiation, and metabolism	Differentiates SLE with or without LN
Chen et al. (2023)	tiRNA5-Lys-CTT-1	Lupus nephritis	Participates in cell signaling and growth regulation	Potential biomarker for LN progression
Ding et al. (2024)	tRF-19-DRMD5112	Renal cell carcinoma	Shows a specifically high abundance in the peripheral blood of RCC patients	Serves as a highly sensitive liquid biopsy marker for early RCC diagnosis
Ding et al. (2024)	tRF-28-87R8WP91IE0K	Renal cell carcinoma	Downregulated in tumor tissues; *in vitro* experiments confirmed its ability to suppress malignant behavior of cancer cells	Has potential as a tumor suppressor and a promising therapeutic target
Kuscu et al. (2021)	tsRNAs	Kidney transplantation	Function as early response molecules to transplantation stress; levels are associated with graft injury and patient outcomes	Enable real-time graft monitoring and prognosis assessment
Williams et al. (2022)	3′-tRFs, 5′-tRFs	Focal segmental glomerulosclerosis	Widely dysregulated in renal tissues, with expression changes preceding morphological injury	Potential early molecular warning signals for FSGS
Luo et al. (2020)	tRF-Val-AAC-007	IgA nephropathy	Upregulated in PBMCs, regulates immunometabolism	Provides insights into the pathogenesis of IgA nephropathy and has potential for disease monitoring

## Limitation

To date, no peer-reviewed studies have directly profiled or functionally characterized tsRNAs (including tRFs/tiRNAs) in polycystic kidney disease (PKD/ADPKD). Existing small RNA studies in PKD have focused primarily on miRNAs and a few piRNAs. For example, several reports have documented altered miR-192/194/30 family members in urinary extracellular vesicles (uEVs) from ADPKD patients, yet tsRNAs were neither analyzed nor reported (Ali et al., 2024; Magayr et al., 2020). Meanwhile, in other kidney diseases such as AKI and CKD, tsRNAs have been reliably detected in urine-derived exosomes, suggesting their feasibility as biomarkers. This discrepancy may partially stem from technical biases: conventional small RNA sequencing often underdetects tsRNAs due to RNA base modifications (*e.g.*, methylation), unless demethylation-enhanced methods like ARM-seq or DM-tRNA-seq are applied (Padhiar et al., 2024). Hence, the current absence of tsRNAs data in PKD may reflect technical underestimation rather than biological absence.

Beyond this, the clinical application of tsRNAs still faces important methodological barriers. tsRNAs frequently undergo extensive post-transcriptional modifications (*e.g.*, m^5^C, Ψ), which hinder their capture by standard sequencing protocols and complicate assay standardization. In addition, the field relies heavily on bioinformatic predictions of target genes and regulatory pathways, with few studies validating these interactions through classic biochemical techniques such as RIP, RNA pull-down, or dual-luciferase reporter assays.

To realize their translational potential, future research must rigorously verify tsRNAs target networks and reduce dependency on computational inference. Well-designed prospective studies are urgently needed to compare tsRNAs markers directly with traditional indicators like serum creatinine, evaluating diagnostic accuracy *via* ROC analysis in real-world settings.

## Summary and Outlook

### tsRNAs and epigenetic crosstalk

Recent advances suggest that tsRNAs can function beyond post-transcriptional regulation, influencing epigenetic processes such as DNA methylation, histone modification, and chromatin remodeling. For instance, specific tRFs interact with DNA methyltransferases (DNMTs), altering global methylation profiles in inflammatory and tumor settings (Park et al., 2020). Notably, Goodarzi et al. (2015) revealed that certain tsRNAs displace YBX1 to suppress endogenous retroelements, suggesting roles in genome defense and chromatin stabilization.

Although this epigenetic layer of regulation has not been extensively studied in kidney diseases, it represents a promising direction for uncovering novel mechanisms of gene regulation under pathological conditions such as hypoxia, inflammation, or fibrosis.

### Future directions and multi-omics integration

Looking forward, integrative multi-omics approaches will be instrumental in advancing tsRNAs research from basic discovery to clinical translation. By combining tsRNAs expression profiles with data from GWAS, epigenomic, transcriptomic, and proteomic analyses, researchers may construct a more precise molecular classification of kidney diseases and identify functionally relevant tsRNA regulators.

For example, tRIP-seq combined with phosphoproteomics can elucidate how tsRNAs dynamically control translation and signal transduction. Large-scale population studies incorporating multiple omics layers may yield robust tsRNAs signatures associated with renal function decline, drug response, or clinical prognosis.

The application of cutting-edge tools such as renal organoids and single-cell multi-omics will further clarify the cell-type-specific roles and spatiotemporal expression patterns of tsRNAs in disease initiation and progression. If successfully implemented, these strategies could elevate tsRNAs from molecular biomarkers to central hubs in renal regulatory networks, ultimately enhancing precision diagnostics and therapeutic targeting in nephrology.

##  Supplemental Information

10.7717/peerj.20315/supp-1 Supplemental Information 1 PRISMA checklist

10.7717/peerj.20315/supp-2 Supplemental Information 2 PRISMA 2020 flow diagram This flow diagram illustrates the study identification, screening, eligibility, and inclusion process.

10.7717/peerj.20315/supp-3 Supplemental Information 3 Supplemental Methods The research methodology of a systematic review, including searching for literature on tsRNAs, tRFs, and tiRNAs related to kidney disease in PubMed and Web of Science databases (as of December 2024), and manually supplementing and screening references. The inclusion criteria are original studies exploring the role of tRNA derived small RNAs in kidney disease, excluding reviews and unrelated literature. Through automated deduplication, independent review, and full-text review processes, 40 studies were ultimately included.

## Additional Information and Declarations

### Data Availability

The following information was supplied regarding data availability: This is a literature review.
