# Supplementary material for: Research progress of tsRNAs in kidney diseases
Source: PeerJ. 2025 Nov 10;13:e20315. doi: 10.7717/peerj.20315 (PMC12614097; doi:10.7717/peerj.20315)
Supplement: Supplemental Information 2 — This flow diagram illustrates the study identification, screening, eligibility, and inclusion process. [file peerj-13-20315-s002.docx]

**Identification of studies via databases and registers**

Records removed *before screening*:

Duplicate records removed (n =0)

Records marked as ineligible by automation tools (n = 0)

Records removed for publication year outside 2014–2025(n = 551)

Records identified from*:

PubMed(n=1440)

Web of Science(n=916)

**Identification**

Records excluded**

(n = 756)

756 were excluded by a human and 1523 excluded by automation tools.

Records screened

(n = 1805)

Reports not retrieved

(n = 0)

Reports sought for retrieval

(n =1236)

**Screening**

Reports excluded:

Non-tsRNAs/tRFs/tiRNAs studies(n=399)

Non-experimental studies (n=112)

Non-renal disease-related studies(n=704)

Reports assessed for eligibility

(n =1236)

Studies included in review

(n =55)

Reports of included studies

(n =72)

*The discrepancy is attributed to supplementary searches and manual adjustments during full-text review.

**Included**

*Consider, if feasible to do so, reporting the number of records identified from each database or register searched (rather than the total number across all databases/registers).

**If automation tools were used, indicate how many records were excluded by a human and how many were excluded by automation tools.

Source: Page MJ, et al. BMJ 2021;372:n71. doi: 10.1136/bmj.n71.

This work is licensed under CC BY 4.0. To view a copy of this license, visit <https://creativecommons.org/licenses/by/4.0/>
