# Supplementary material for: Research progress of tsRNAs in kidney diseases
Source: PeerJ. 2025 Nov 10;13:e20315. doi: 10.7717/peerj.20315 (PMC12614097; doi:10.7717/peerj.20315)
Supplement: Supplemental Information 3 — The research methodology of a systematic review, including searching for literature on tsRNAs, tRFs, and tiRNAs related to kidney disease in PubMed and Web of Science databases (as of December 2024), and manually supplementing and screening references. The inclusion criteria are original studies exploring the role of tRNA derived small RNAs in kidney disease, excluding reviews and unrelated literature. Through automated deduplication, independent review, and full-text review processes, 40 studies were ultimately included. [file peerj-13-20315-s003.docx]

**Supplemental Material: Methods Details**

**1. Search Strategy**

Full search strategies (including Boolean operators and filters) for each database:

tsRNAs or tRFs or tiRNAs and kidney disease

**2. Information Sources**

- **Databases**: PubMed, Web of Science.
- **Search Date**: All searches were conducted up to 31 December 2024
- **Supplementary Sources**: Reference lists of included studies and relevant reviews were manually screened.

**3. Eligibility Criteria**

Studies were included if they:

- Investigated the role of **tsRNAs, tRFs, tiRNAs, or tRNA-derived fragments** in the context of **kidney disease** (including acute kidney injury, chronic kidney disease, diabetic nephropathy, etc.).
- Were original research articles (e.g., experimental, observational, or clinical studies).

Exclusion criteria:

- Reviews, Meta-Analysis，Systematic Review，Editorials.
- Studies lacking direct relevance to tRNA-derived fragments or kidney disease.

**4. Selection Process**

1. **Automated Screening**: All retrieved records were imported into **Rayyan** for deduplication and initial title/abstract screening.
2. **Independent Screening**: Two reviewers (initials: **Jialing Wang**and **Yanzhe Wang**) independently screened titles/abstracts against inclusion criteria.
3. **Conflict Resolution**: Disagreements were resolved through discussion or consultation with a third reviewer (initials:**Fengqin Li**).
4. **Full-Text Review**: Potentially eligible studies underwent full-text assessment by the same two reviewers.


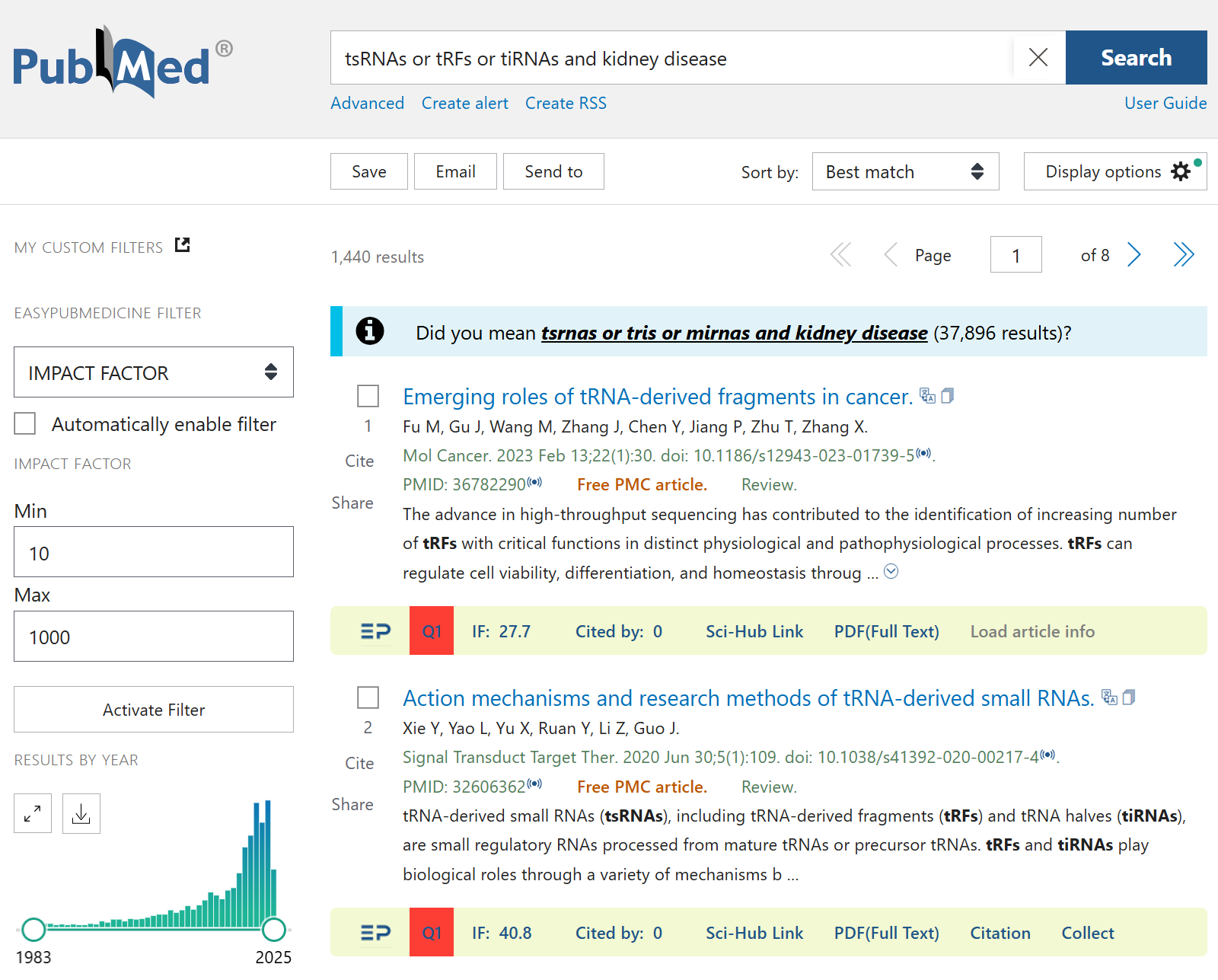


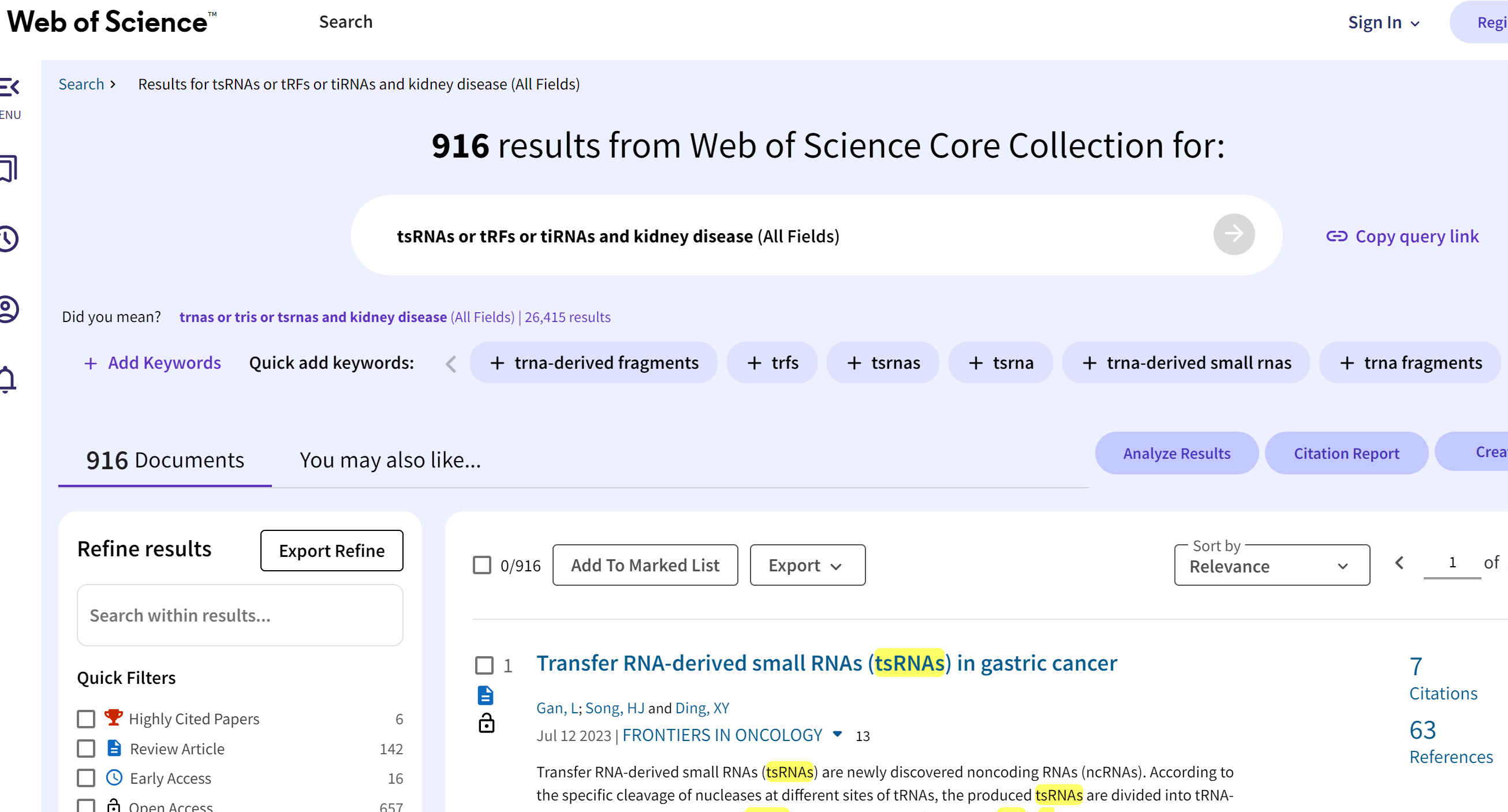


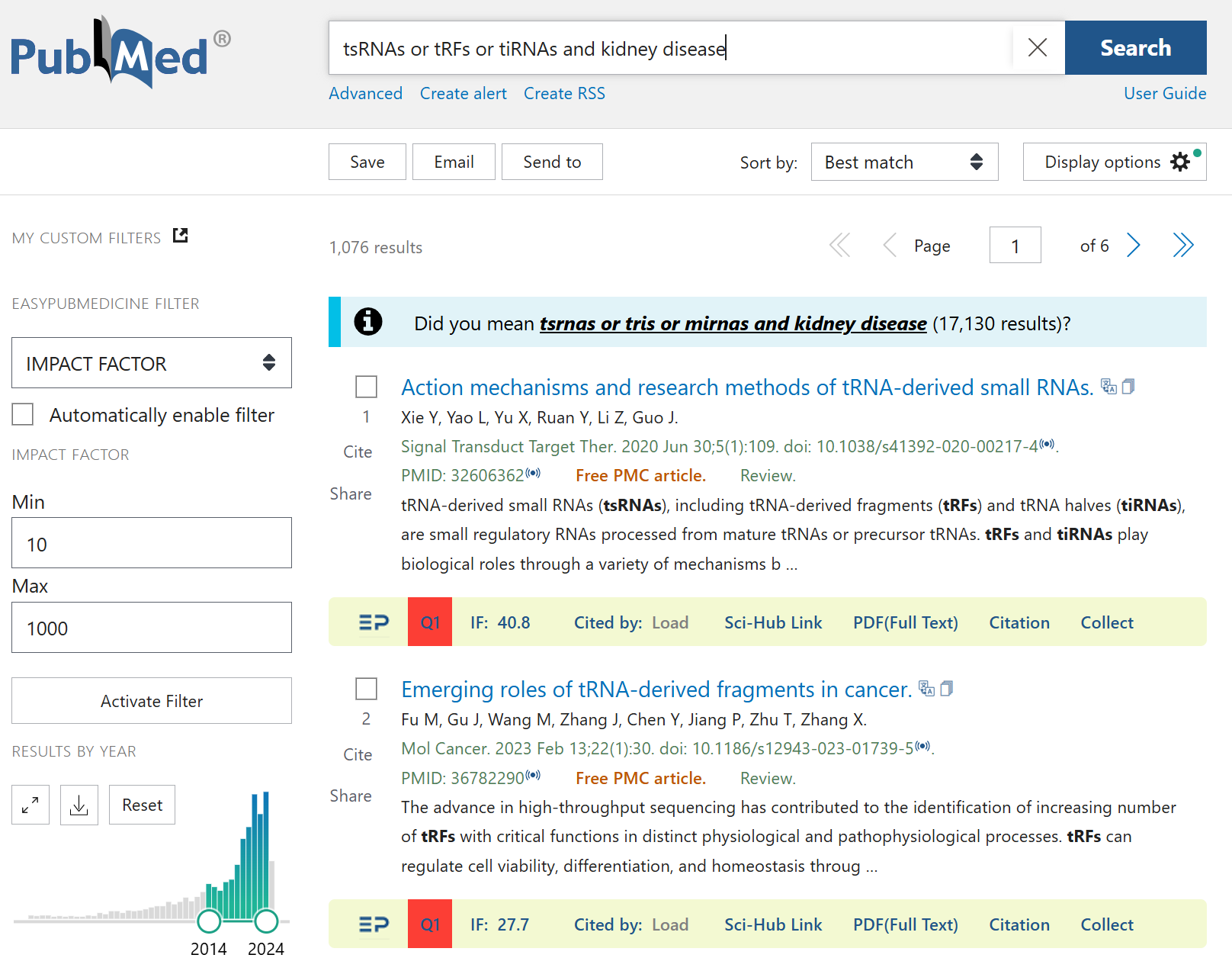


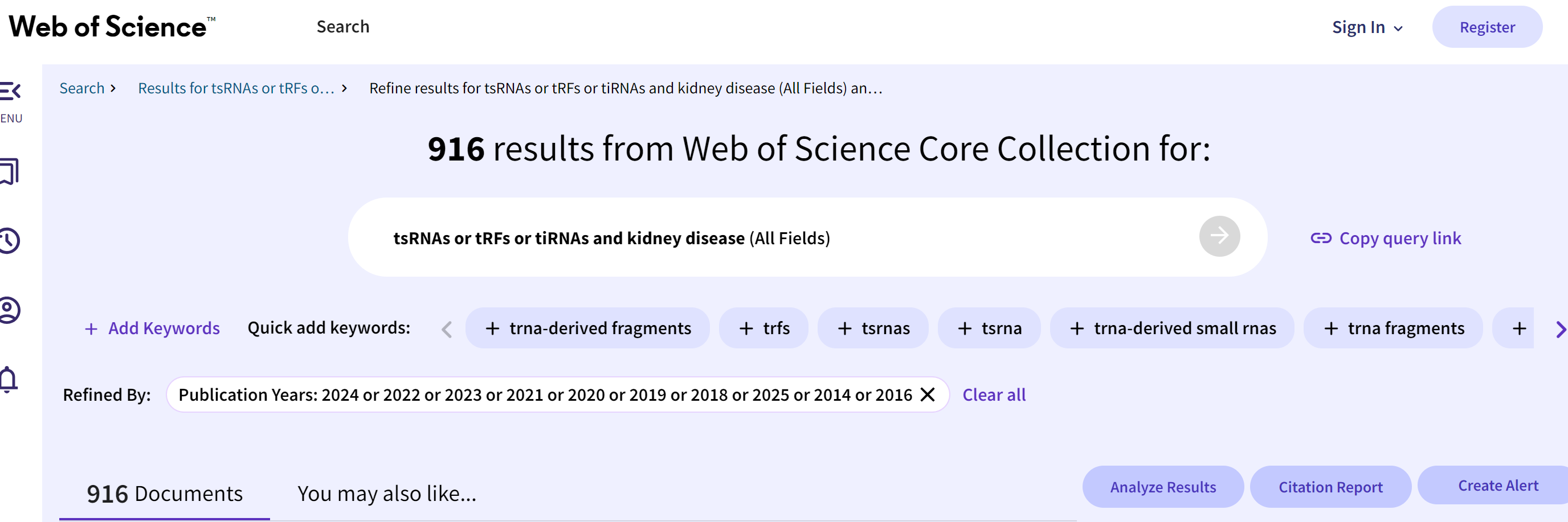


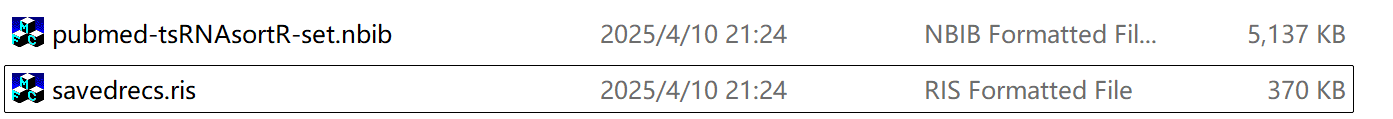


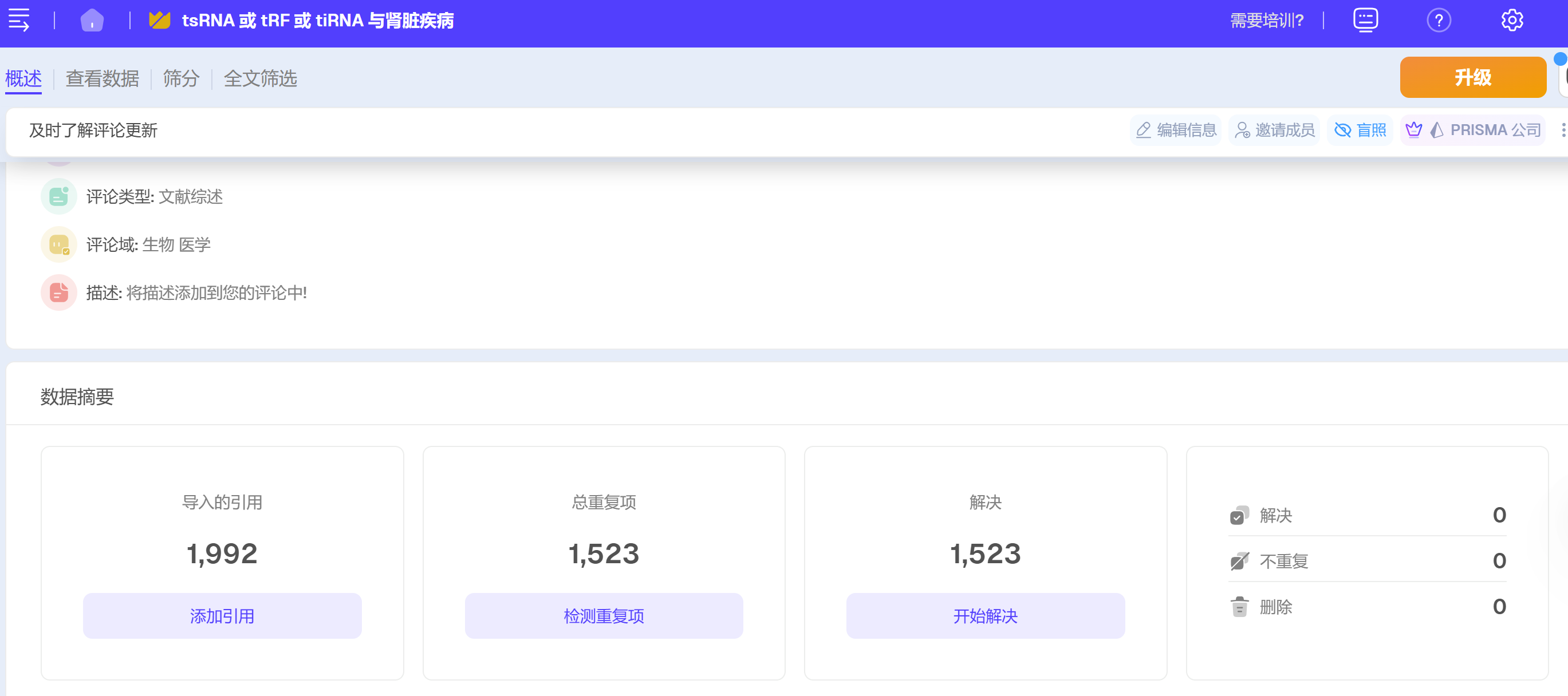


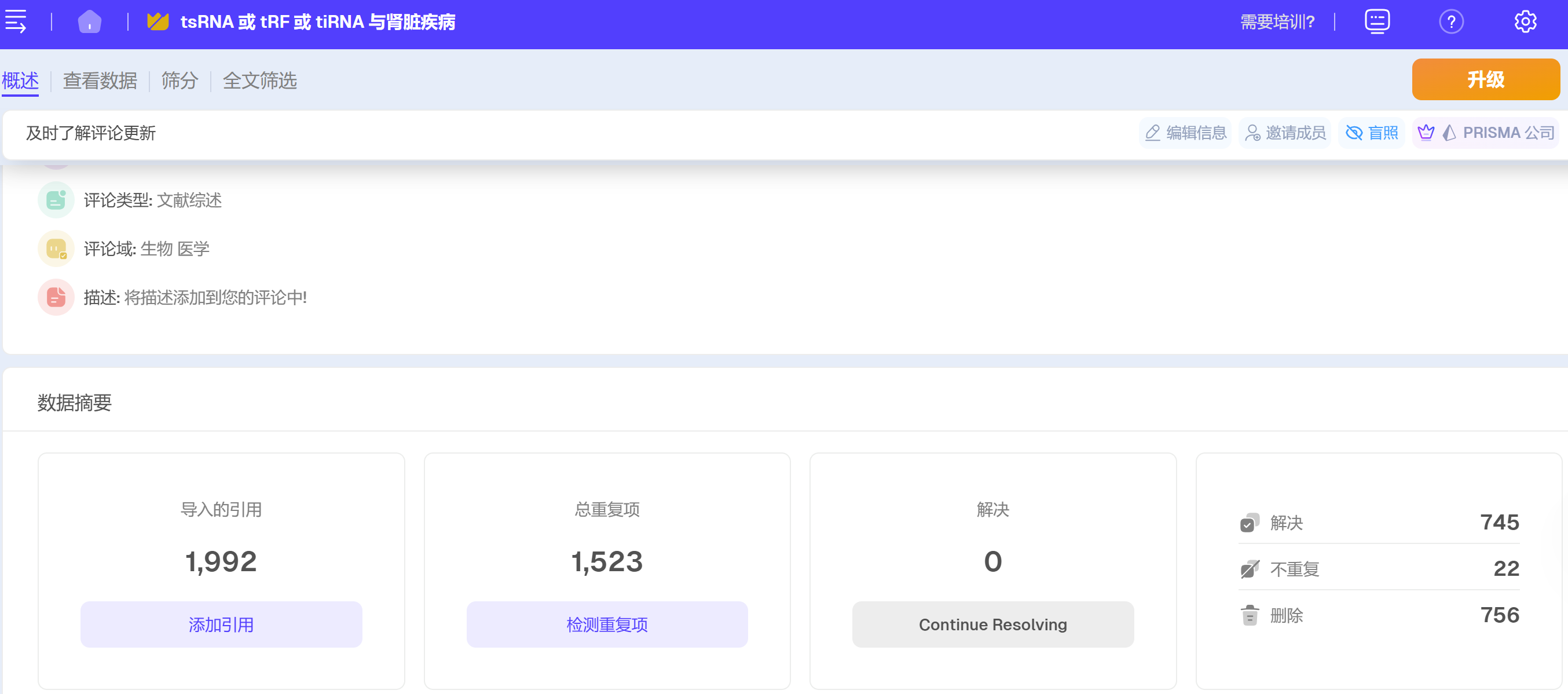


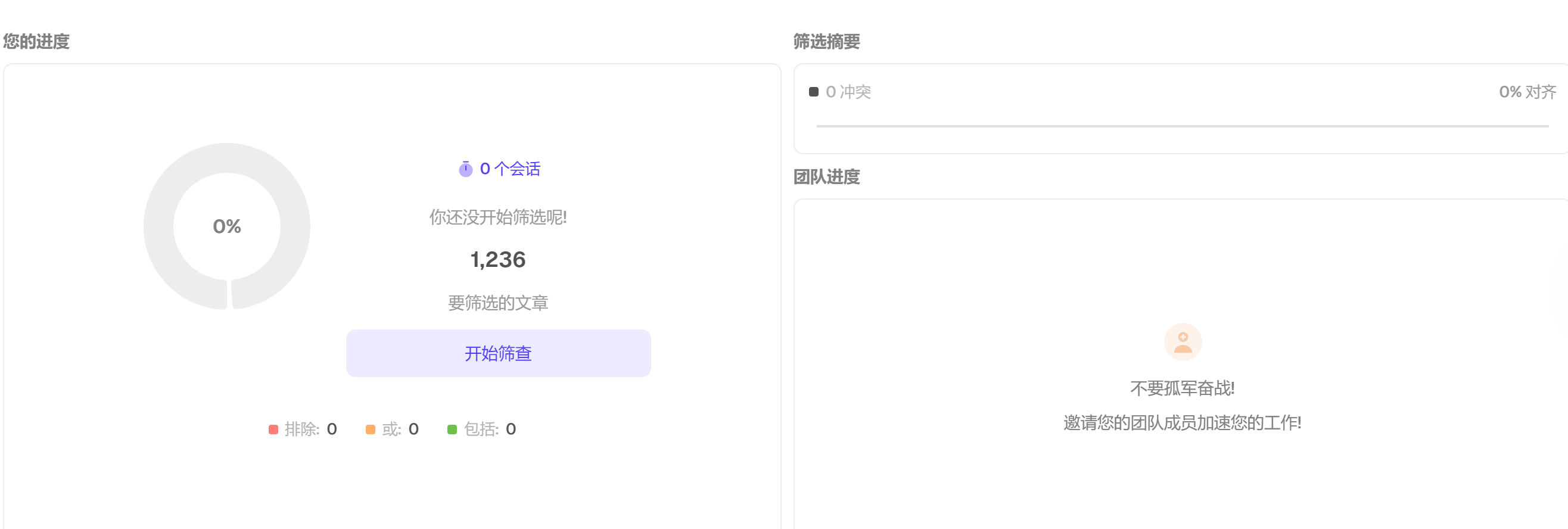


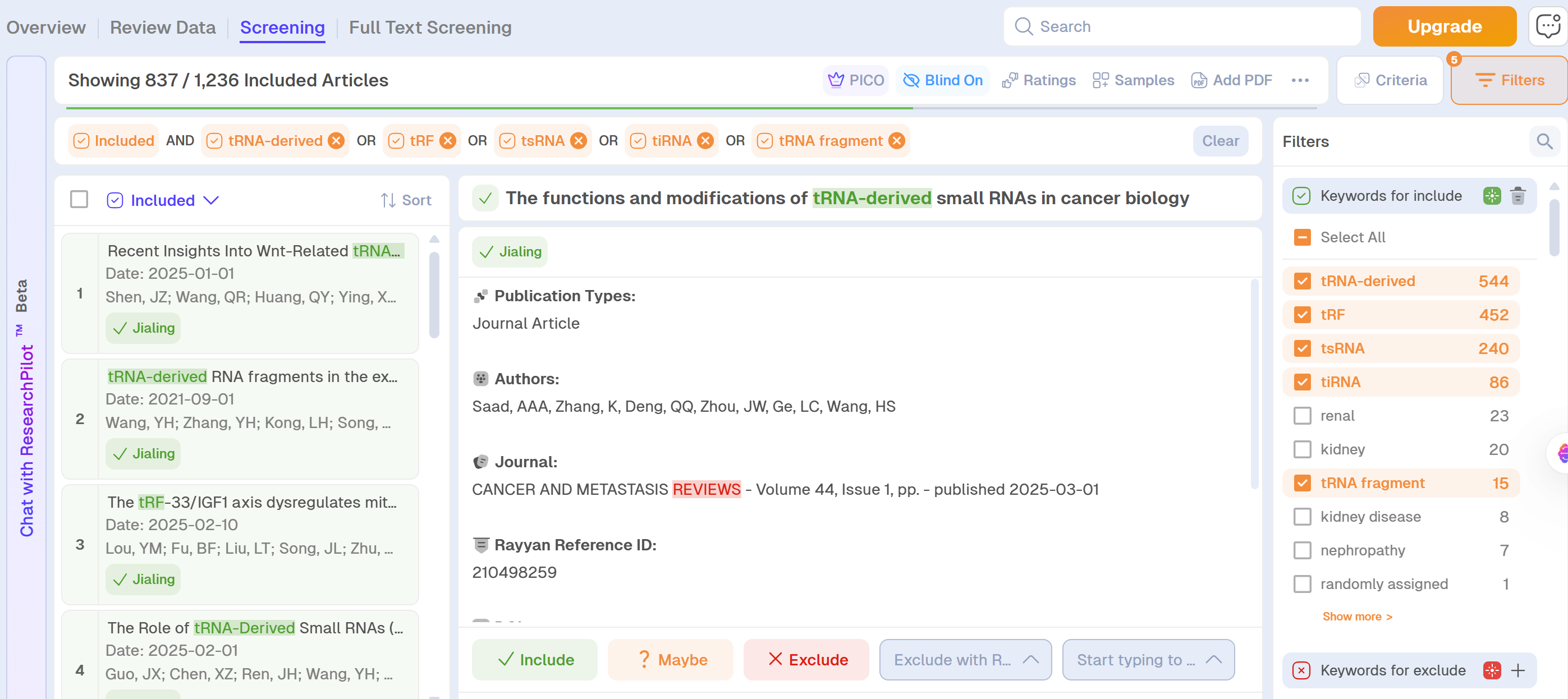


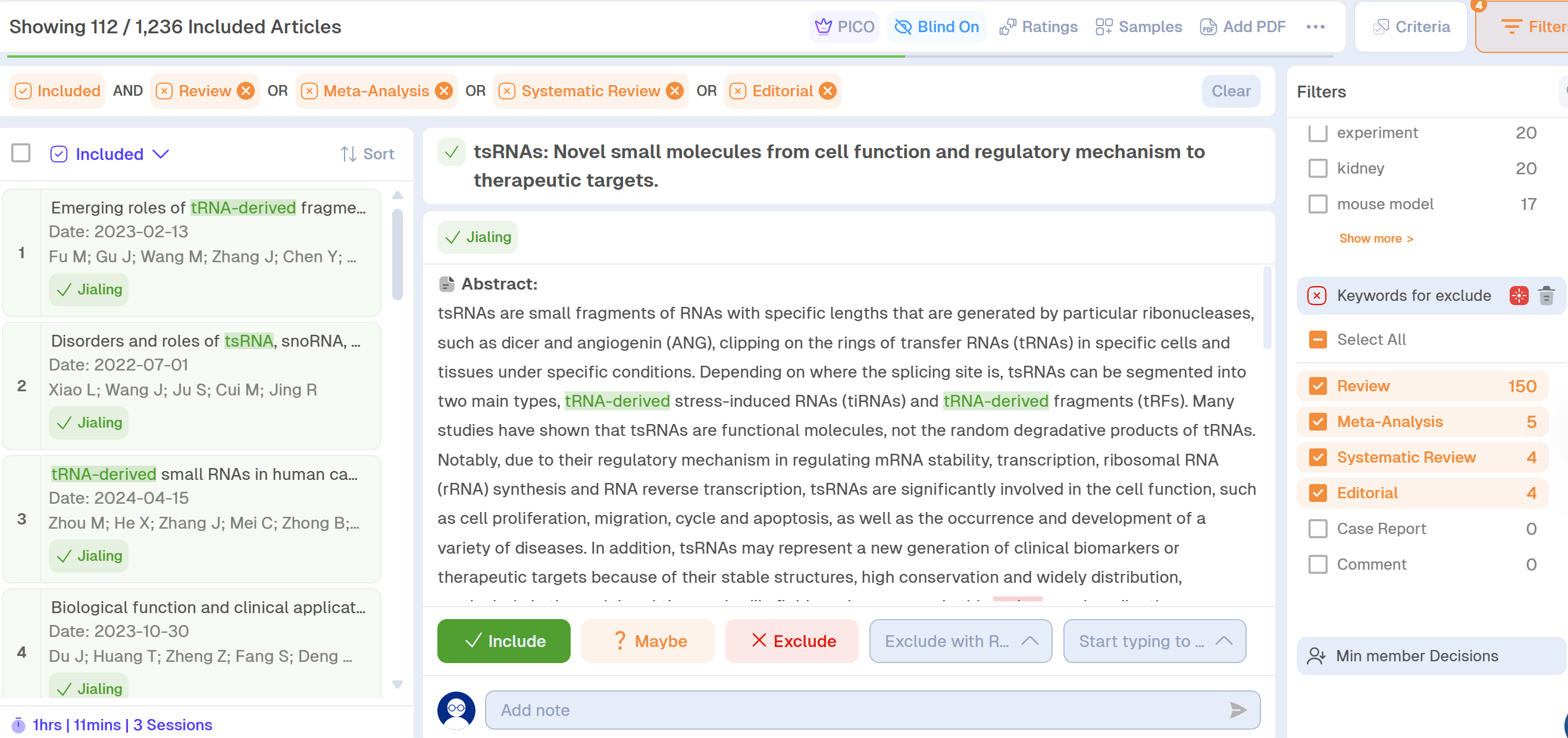


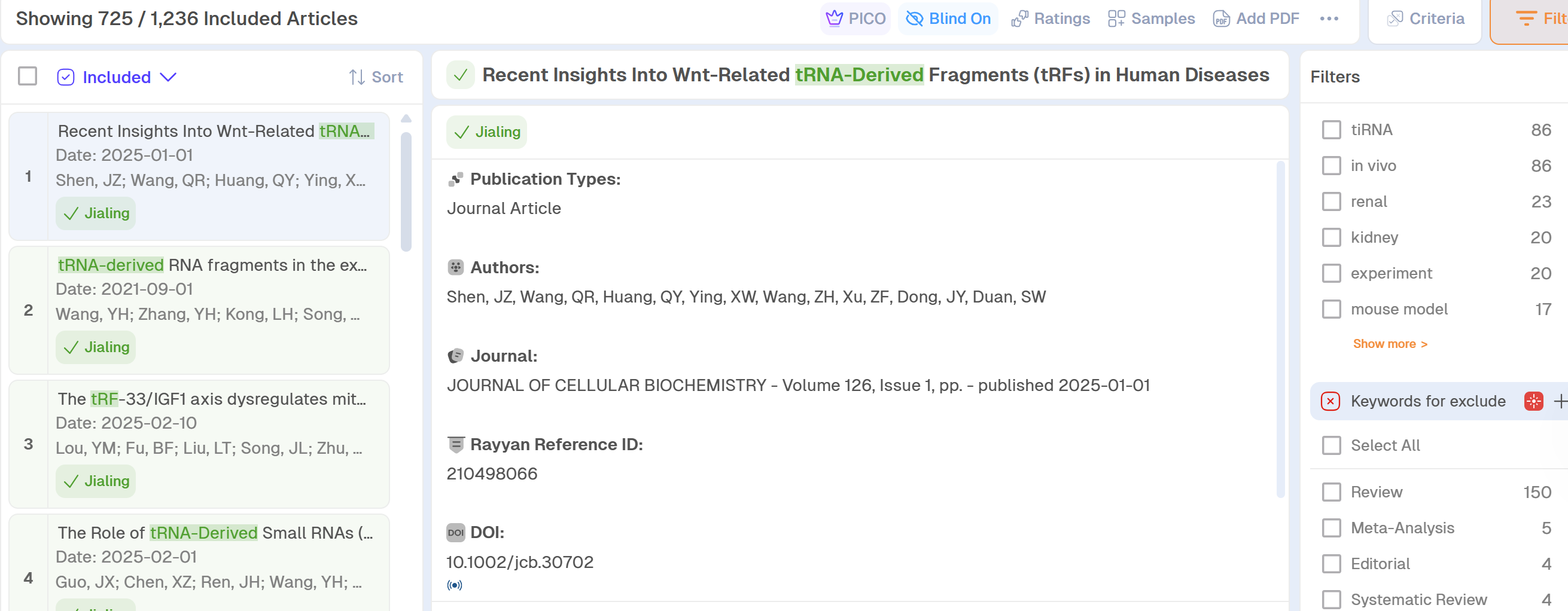


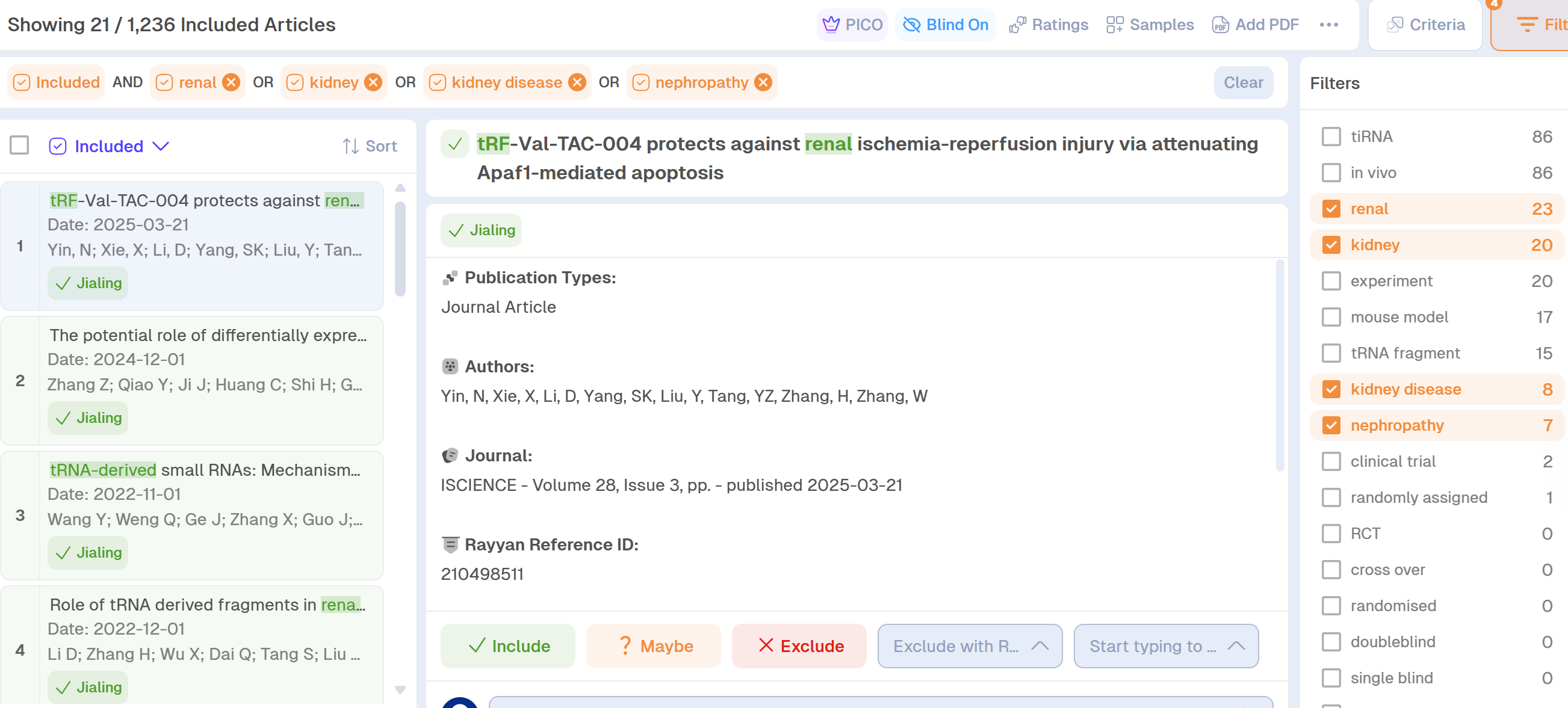


* **Statement:**

Among the 40 cited references, 32 were retrievable in both PubMed and Web of Science, resulting in reports of included studies (n = 72).
